
JOURNAL INFORMATION
==============================
NLM Title Abbreviation: ScientificWorldJournal
Journal ID: ScientificWorldJournal
NLM Journal ID: 101131163
Journal ID: TSWJ

The Scientific World Journal

ISSN: 2356-6140
EISSN: 1537-744X
Publisher: Wiley

ARTICLE INFORMATION
==============================
PMCID: PMC6009369
PMID: 12806026
DOI: 10.1100/tsw.2002.122
Article ID: 950120
Article version: 1
Subjects: Short Review

Soluble HLA Class I Molecules/CD8 Ligation Trigger Apoptosis of CD8 Cells by Fas/Fas-Ligand Interaction

Puppo Francesco *
Contini Paola
Ghio Massimo
Indiveri Francesco
Department of Internal Medicine (DIMI), Viale Benedetto XV, n. 6, 16132 Genoa, Italy
*Francesco Puppo: metclin@unige.it
Publication date: 2002
Electronic publication date: 2002 Feb 12
Volume: 2
First page: 421
Last page: 423
Received 2001 Nov 29; Accepted 2002 Jan 4
Copyright: Copyright © 2002 Francesco Puppo et al.
Copyright year: 2002
License: This is an open access article distributed under the Creative Commons Attribution License, which permits unrestricted use, distribution, and reproduction in any medium, provided the original work is properly cited.
License URL: https://creativecommons.org/licenses/by/3.0/

Keywords: soluble HLA, apoptosis, Fas ligand, CD8

==============================
Short Review
*TheScientificWorldJOURNAL* (2002) 2, 421–423
ISSN 1537-744X; DOI 10.1100/tsw.2002.122

### Soluble HLA Class I Molecules/CD8 Ligation Trigger Apoptosis of CD8$^+$ Cells by Fas/Fas-Ligand Interaction

Francesco Puppo*, Paola Contini, Massimo Ghio, and Francesco Indiveri
*Department of Internal Medicine (DIMI), Viale Benedetto XV, n. 6, 16132 Genoa, Italy*

*Received November 29, 2001; Accepted January 4, 2002; Published February 12, 2002*

**KEY WORDS:** soluble HLA, apoptosis, Fas ligand, CD8

**DOMAINS:** inflammation, cell cycle (fate), hematology

The human major histocompatibility complex (HLA) encodes two sets of HLA class I molecules, which have been termed class Ia (or classical) and class Ib (or nonclassical) molecules. The class Ia molecules include the gene products of HLA-A, HLA-B, and HLA-C loci and are characterized by broad tissue expression and by a high degree of polymorphism. The class Ib molecules include the gene products of HLA-E, HLA-F, and HLA-G loci and are characterized by a restricted tissue distribution and by limited polymorphism. Besides being expressed on nucleated cells, classical and nonclassical HLA class I molecules are present in serum in soluble form (sHLA-I)[1,2]. The serum level of sHLA-I molecules is significantly increased in a variety of physiological and pathological conditions such as pregnancy, acute rejection episodes following organ allografts, acute graft-versus-host-disease (GVHD) following bone marrow transplantation, autoimmune diseases, viral infections, and malignant melanoma[3,4,5,6]. Because of the statistically significant association with clinical parameters, the level of sHLA-I antigens has been suggested to represent a useful marker to predict the evolution of viral infections and to monitor the clinical course of allografts[7]. Moreover, elevated levels of functional sHLA-I and soluble Fas-ligand molecules have been detected by our group in blood components and might play a role in the immunomodulatory effect of autologous and allogeneic transfusions[8,9,10,11]. Several lines of evidence suggest that sHLA-I molecules are immunologically functional and may play an immunoregulatory role. In fact, they have been shown to elicit antibodies in both allogeneic and xenogeneic combinations, to inhibit the activity of alloreactive cytotoxic T lymphocytes (CTL)[12,13,14], and to induce apoptosis in alloreactive and virus-specific CTL, in activated autologous and allogeneic CD8$^+$ T cells, and in CD8$^+$ NK cells[15,16,17,18,19]. There is general agreement about the mechanism underlying the inhibition of CTL activity by sHLA antigens. This inhibition appears to be mediated by interactions of sHLA-I antigens $\alpha_1$ and $\alpha_2$ domains with T cell receptor (TCR)[12,13,14]. By contrast, there is conflicting information about the mechanism underlying induction of apoptosis of activated T cells by sHLA-I antigens. Several authors reported that sHLA-I molecules induced apoptosis of alloreactive CD8$^+$ cytotoxic T lymphocytes through interaction with their TCR[15]. However, our own data and those other groups indicate that classical and nonclassical sHLA-I molecules trigger

*Corresponding author. Email: metclin@unige.it
©2002 with author.

Fas/Fas-ligand mediated apoptosis of phytohemoagglutinin (PHA)-activated and virus-specific $CD8^+$ T lymphocytes as well as of $CD8^+$ NK cells by interacting with CD8 coreceptor[17,18,19,20]. Recently, we performed a series of experiments in our laboratory to clarify the intracellular mechanism(s) leading to Fas-ligand upregulation and secretion. These unpublished data indicate that sHLA-I/CD8 ligation elicits the phosphorylation of $p56^{lck}$ protein thyrosin kinase (PTK) associated with CD8 cytoplasmic domain in the absence of any other TCR-derived signal, the activation of *syk*-like ZAP-70 PTK and protein kinase C, and extracellular calcium influx. Then, activation and nuclear translocation of NF-kB and NF-AT occurs, leading to Fas-ligand mRNA transcription and soluble Fas-ligand secretion, which delivers the death signal. Interestingly, soluble Fas-ligand secretion and $CD8^+$ cell apoptosis, but not $CD8^+$ cell cytolitic activity, are completely inhibited by Cyclosporin A, which specifically blocks the activation of the calcineurin/calmodulin pathway. Taken together, these data suggest that sHLA-I molecules are involved in a signal-transduction pathway leading to Fas-ligand expression, soluble Fas-ligand secretion, and $CD8^+$ cells apoptosis.

Apoptosis induced by Fas/Fas-ligand interactions plays a crucial role for the establishment of antigen-specific T cell tolerance, both during the intrathymic negative selection and during adult life[21,22,23,24,25]. The amount of sHLA-I molecules that induces apoptosis in $CD8^+$ cells is analogous to the level found in plasma of patients with an activation of their immune system. Therefore, sHLA-I antigens secreted during immune system activation may bind to CD8 molecules on activated $CD8^+$ cells and induce soluble Fas-ligand secretion. Then, soluble Fas-ligand may act in an autocrine and/or paracrine way, triggering apoptosis in activated $CD8^+$ $CD95^+$ cells. If so, serum sHLA-I molecules may represent an important efferent arm of the network to control the expansion of $CD8^+$ cells and to downregulate immune responses and could be proposed as potential immunosuppressive tools in the field of transplantation and autoimmunity.

### REFERENCES

1. Van Rood, J.J., van Leeuwen, A., and van Santen, M.C.T. (1970) Anti HL-A2 inhibitor in normal human serum. *Nature* **226**, 366–367.
2. Charlton, R.K. and Zmijewski, C.M. (1970) Soluble HL-A7 antigen: localization in the β-lipoprotein fraction of human serum. *Science* **170**, 636–637.
3. Puppo, F., Brenci, S., Lanza, L., Bosco, O., Imro, M.A., Scudeletti, M., Indiveri, F., and Ferrone, S. (1994) Increased level of serum HLA Class I antigens in HIV infection. Correlation with disease progression. *Hum. Immunol.* **40**, 259–266.
4. Puppo, F., Scudeletti, M., Indiveri, F., and Ferrone, S. (1995) Serum HLA class I antigens: markers and modulators of an immune response? *Immunol. Today* **16**, 124–127.
5. Hunt, J.S., Jadhav, L., Chu, W., Geraghty, D.E., and Ober, C. (2000) Soluble HLA-G circulates in maternal blood during pregnancy. *Am. J. Obstet. Gynecol.* **183**, 682–688.
6. Ugurel, S., Rebmann, V., Ferrone, S., Tilgen, W., Grosse-Wilde, H., and Reinhold, U. (2001) Soluble human leukocyte antigen-G serum level is elevated in melanoma patients and is further increased by interferon-alpha immunotherapy. *Cancer* **92**, 369–376.
7. Puppo, F., Indiveri, F., Scudeletti, M., and Ferrone, S. (1997) Soluble HLA antigens: new roles and uses. *Immunol. Today* **18**, 154–155.
8. Ghio, M., Contini, P., Mazzei, C., Brenci, S., Barberis, G., Filaci, G., Indiveri, F., and Puppo F. (1999) Soluble HLA class I, HLA class II and Fas ligand in blood components: a possible key to explain the immunomodulatory effects of allogeneic blood transfusions. *Blood* **93**, 1770–1777.
9. Ghio, M., Contini, P., Mazzei, C., Brenci, S., Filaci, G., Indiveri, F., and Puppo, F. (2000) Soluble HLA class I and Fas ligand molecules in blood components and their role in the immunomodulatory effects of blood transfusions. *Leuk. Lymphoma* **37**, 1–8.
10. Puppo, F., Ghio, M., Contini, P., Mazzei, C., and Indiveri F. (2001) Fas, Fas ligand, and transfusion immunomodulation. *Transfusion* **41**, 416–418.
11. Ghio, M., Contini, P., Mazzei, C., Merlo, A., Filaci, G., Setti, M., Indiveri, F., and Puppo, F. (2001) In vitro immunosuppressive activity of soluble HLA class I and Fas ligand molecules: do they play a role in autologous blood transfusion? *Transfusion* **41**, 988–996.

12. Hausmann, R., Zavazava, N., Steinmann, J., and Müller-Ruchholtz, W. (1993) Interaction of papain-digested HLA class I molecules with human alloreactive cytotoxic T lymphocytes (CTL). *Clin. Exp. Immunol.* **91**, 183–188.
13. Parham, P., Clayberger, C., Zorn, S.L., Ludwig, D.S., Schoolnik, G.K., and Krensky, A.M. (1987) Inhibition of alloreactive cytotoxic T lymphocytes by peptides from the $\alpha_2$ domain of HLA-A2. *Nature* **325**, 625–628.
14. Dal Porto, J., Johansen, T.E., Catipovic, B., Parfiit, D.J., Tuveson, D., Gether, U., Kozlowski, S., Fearon, D.T., and Schneck, J.P. (1993) A soluble divalent class I major histocompatibility complex molecule inhibits alloreactive T cells at nanomolar concentrations. *Proc. Natl. Acad. Sci. U. S. A.* **90**, 6671–6675.
15. Zavazava, N. and Krönke, M. (1996) Soluble HLA class I molecules induce apoptosis in alloreactive cytotoxic T lymphocytes. *Nat. Med.* **2**, 1005–1010.
16. Contini, P., Ghio, M., Merlo, A., Brenci, S., Filaci, G., Indiveri, F., and Puppo, F. (2000) Soluble HLA class I/CD8 ligation triggers apoptosis in EBV-specific CD8+ cytotoxic T lymphocytes by Fas/Fas-ligand interaction. *Hum. Immunol.* **61**, 1347–1351.
17. Gansuvd, B., Haghiara, M., Munkhbat, B., Kanai, N., Morita, N., Munkhtuvshin, N., Chargui, J., Kato, S., Hotta, T., and Tsuji, K. (2000) Inhibition of Epstein-Barr virus (EBV)-specific $CD8^+$ cytotoxic T lymphocyte (CTL) activity by soluble HLA class I in vitro. *Clin. Exp. Immunol.* **119**, 107–114.
18. Puppo, F., Contini, P., Ghio, M., Brenci, S., Scudeletti, M., Filaci, G., Ferrone, S., and Indiveri, F. (2000) Soluble human MHC class I molecules induce soluble Fas ligand secretion and trigger apoptosis in activated $CD8^+$ $Fas(CD95)^+$ T lymphocytes. *Int. Immunol.* **12**, 195–203.
18. Spaggiari, G.M., Contini, P., Carosio, R., Arvigo, M., Ghio, M., Oddone, D., Dondero, A., Zocchi, M.R., Puppo, F., Indiveri, F., and Poggi, A. (2001) Soluble HLA class I molecules induce Natural Killer cell apoptosis through the engagement of CD8. Evidence for a negative regulation exerted by CD94/NKG2A complex and KIR2D. *Blood*, in press.
20. Fournel, S., Aguerre-Girr, M., Huc, X., Lenfant, F., Alam, A., Toubert, A., Bensussan, A., and Le Bouteiller, P. (2000) Soluble HLA-G1 triggers CD95/CD95 ligand-mediated apoptosis in activated $CD8^+$ cells by interaction with CD8. *J. Immunol.* **164**, 6100–6104.
21. Mountz, J.D., Zhou, T., Wu, J., Wang, W., Su, X., and Cheng, J. (1995) Regulation of apoptosis in immune cells. *J. Clin. Immunol.* **15**, 1–16.
22. van Parijs, L. and Abbas, A.K. (1996) Role of Fas-mediated cell death in the regulation of immune responses. *Curr. Opin. Immunol.* **8**, 355–361.
23. Yonehara, S., Nishimura, Y., Kishil, S., Yonehara, M., Takazawa, K., Tamatani, T., and Ishii, A. (1994) Involvment of apoptosis antigen Fas in clonal deletion of human thymocytes. *Int. Immunol.* **6**, 1849–1856.
24. Lynch, D.H., Ramsdell, F., and Alderson, M.R. (1995) Fas and FasL in the homeostatic regulation of immune responses. *Immunol. Today* **16**, 569–574.
25. Kurts, C., Heath, W.R., Kosaka, H., Miller, J.F.A.P., and Carbone, F. (1998) The peripheral deletion of autoreactive $CD8^+$ T cells induced by cross-presentation of self-antigens involves signaling through CD95 (Fas, Apo-1). *J. Exp. Med.* **188**, 415–420.

**This article should be referenced as follows:**

Puppo, F., Contini, P., Ghio, M., and Indiveri, F. (2002) Soluble HLA class I molecules/CD8 ligation trigger apoptosis of $CD8^+$ cells by Fas/Fas-ligand interaction. *TheScientificWorldJOURNAL* **2**, 421–423.

**Handling Editor:**

Charles Serhan, Principal Editor for *Inflammation* — a domain of *TheScientificWorldJOURNAL*.
